# Digital Removable Denture Workflows in Dental Education: A Systematic Review and Curriculum Development Exploration

**DOI:** 10.1111/eje.70001

**Published:** 2025-06-17

**Authors:** Arthi Veerasamy, Fatimah Ghuloum, Yunam Lo, Wendy Jansen van Vuuren

**Affiliations:** ^1^ Faculty of Dentistry University of Otago Dunedin New Zealand

**Keywords:** CAD/CAM complete denture, dental education, dental schools, dental students, digital complete denture, digital denture workflow

## Abstract

**Background:**

In the realm of dental education, the conventional methods of complete denture fabrication have long been the norm. However, these methods often struggle to effectively communicate complex techniques, leaving students lacking comprehensive hands‐on experiences. Digitalisation offers a potential solution, promising improved accuracy and a revolutionised curriculum. However, low global adoption rates and a lack of established teaching methodologies necessitate investigation into the challenges and successes of integrating digital removable denture workflows.

**Aim:**

This systemic review aims to investigate the integration of digital workflows into removable denture curricula within dental institutions globally.

**Method:**

A modified PICO framework guided the literature search focusing on digital removable denture workflows in dental education. A manual search of English‐language publications from 2010 to 2024 was conducted, selecting studies meeting pre‐defined inclusion criteria. The PRISMA guidelines were followed, including a two‐stage independent screening process by two reviewers to assess eligibility.

**Results:**

The review identified six dental institutions incorporating digitalisation into their removable denture curricula. They utilise 3D‐simulation tools for didactic training, while clinical training favours a hybrid approach. Students reported positive experiences, including enhanced learning efficiency, but voiced concerns about technical issues and aesthetic evaluations. Educators noted improvements in assessment quality and student motivation.

**Conclusions:**

Digital workflows offer substantial educational advantages, but their integration faces challenges including cost, infrastructure limitations and technological hurdles. Further research and development are crucial to overcome these barriers and establish standardised digital denture education across global dental institutions.

## 1 Introduction

Complete edentulism impairs individuals physically and psychologically, affecting essential functions and quality of life [1]. Oral rehabilitation through removable dentures improves oral health‐related quality of life [2]. While removable dentures can be fabricated with labour‐intensive analogue technologies, computer‐aided design and manufacturing (CAD/CAM) have simplified their fabrication and are preferred over conventional workflows [3].

Digital workflow includes data acquisition, processing, planning and fabrication [4]. Data acquisition uses digital scanners to capture patient information either directly or indirectly. The direct approach uses intraoral scanners, while the indirect approach involves scanning impressions or models into 3D‐digital data with CAD software [5]. Data processing and planning merge digital datasets to create precise 3D‐images [6]. Practitioners then use CAD software to design the denture and virtually evaluate fit, function and aesthetics [7]. The finalised design is sent to CAM systems for fabrication using 3‐dimensional (3D) printing or Computerised Numerical Control (CNC) milling [8].

The literature suggested numerous benefits for digital dentures over conventional workflows. Digital workflows streamline production, reducing time and labour and minimising human errors. It is estimated that each conventionally fabricated denture requires 3.5 h more clinical time than digitally fabricated ones [9]. Moreover, it requires fewer steps than the conventional workflow, which reduces the risk of introducing human errors in each step affecting the final accuracy of the denture [10]. Also, a systematic review by Zandinejad et al. concluded that digital dentures fabricated by milling or 3D‐printing techniques have better adaptation and retention compared to conventional ones [11, 12, 13].

Conventional denture methods have a robust body of evidence supporting their efficacy and reliability [14]. Dental professionals are highly skilled in conventional techniques due to extensive training, making them comfortable and efficient with these methods, which are staples in dental curricula [15]. Conventional dentures are often less expensive to produce due to lower initial investments in technology and training. Materials and equipment for conventional fabrication are widely available and familiar to most dental labs, making them easier to access and maintain [16]. However, digital workflow requires practitioners to consider their expertise and comfort with digital tools, substantial training and adjustment to digital‐centric clinical processes [17]. As a result, the successful integration of digital workflows into practice depends on practitioners' technological proficiency, adaptability and readiness to embrace new systems [18].

The gradual adoption of digital dentures in dental education has been notable but remains nascent globally. Loma Linda University integrates digital complete dentures into its curriculum's theoretical and practical components [19]. Recent surveys of U.S. dental schools show a dramatic increase in incorporating digital dentures into their curricula compared to previous years [20, 21, 22]. This trend reflects growing recognition of digital technology's importance in contemporary dental education.

Nevertheless, varying global adoption rates underscore the need for a nuanced understanding of factors hindering the integration of digital dentures into dental curricula. Presently, research into teaching CAD/CAM technologies, workflow processes and hands‐on training varies widely in detail and scope. Despite some publications documenting the adoption of modern technologies by individual dental schools, comprehensive guidelines are lacking about how widely these technologies are being integrated into dental programmes or to what extent students are gaining experience with them.

This systematic review (SR) will examine how dental institutions worldwide integrate digital workflows into curricula. It aims to clarify educational outcomes associated with these integrations and propose curriculum development advancements. This SR will offer strategic insights into enhancing dental education's efficacy in the digital age, the current landscape and identify barriers to integration of digital denture teaching in dental curricula.

## 2 Methods and Materials

This SR addressed the research questions using a modified Patient or Population, Intervention, Control, comparison and Outcome (PICO) approach. Two questions were formulated: First, how is the digital removable denture workflow being integrated into the dental curriculum? Second, what are the educational impacts of this integration on students and educators? The comparison component was omitted since the study did not focus on contrasting the digital denture workflow with the conventional workflow. The preferred reporting items for Systematic Reviews and Meta‐Analyses (PRISMA) guidelines are followed throughout the process to ensure transparency and robust reporting [23].

### 2.1 Search Strategy

Utilised included Medline via Ovid, Embase via Ovid, Scopus via Elsevier, PubMed and Web of Science, all of which are recognised as authoritative sources within the medical and scientific communities. In the Medline and Embase databases, the search employed a combination of controlled vocabulary (medical subject headings) and free‐text terms. Other databases were searched using targeted keywords located in article titles, abstracts, and designated keywords (see Supporting Information for specific search terms). To refine the search results further, specific restrictions were applied. Filters were set for “English language” to enhance accessibility and comprehension, and a date range restriction was imposed, focusing on articles published ‘from 2010 to present’. This approach ensured that the study emphasised the most current research and advancements in the field.

### 2.2 Study Selection

The literature selection process followed the steps outlined in the PRISMA 2020 flow diagram, with a focus on the number of records at each step and the reasons for their exclusion. This involved an initial identification of records through database searching, followed by a two‐stage screening process to exclude irrelevant studies based on the selection criteria presented in Table 1, culminating in the final inclusion of studies.

**TABLE 1:** Selection criteria.

Criterion	Inclusion	Exclusion
Population	Dental students, dental educators	Not involving dental students
Setting	Dental educational institutions globally	Non‐dental educational institutions, for example, private practices or hospitals
Topic	Dental curriculum on digital removable prostheses including complete denture and partial denture	Focused on conventional denture only; fixed prosthodontics such as overdenture, implant, crown and bridge; other aspects of dentistry such as orthodontic appliances
Study designs	Observational studies, survey, randomised controlled trial, crossover trial	Review articles
Publication type	Peer‐reviewed, full text original scientific research articles	Editorials and opinion pieces; conference presentations; abstracts only
Language	English	Non‐English
Dates	Published between 2010 and 2024	Published before 2010

### 2.3 Data Extraction

The selected articles then underwent data extraction to collect information on the author's name, year of publication, study design, sample size, study setting, length of study, study aim and details regarding curriculum design of digital removable dentures and educational impacts of this integration. To ensure inter‐rater reliability, two reviewers conducted the study selection and data extraction processes independently. Disagreements between reviewers were resolved by discussion and consensus. Where necessary, a third of the reviewers were involved.

### 2.4 Quality Assessment

The methodological quality of each selected study was evaluated using the Critical Appraisal tools from the Joanna Briggs Institute (JBI). JBI offers various checklists tailored to specific study designs, making them suitable for systematic review that may encounter both qualitative and quantitative studies. These checklists contain questions aimed at evaluating how well a study has addressed the potential for bias at its design and outcome level [24]. The assessment of each study was conducted by two reviewers independently. Decisions regarding the inclusion of each study were made through discussion, with a third reviewer involved only if a consensus could not be reached.

## 3 Results

**FIGURE 1:**
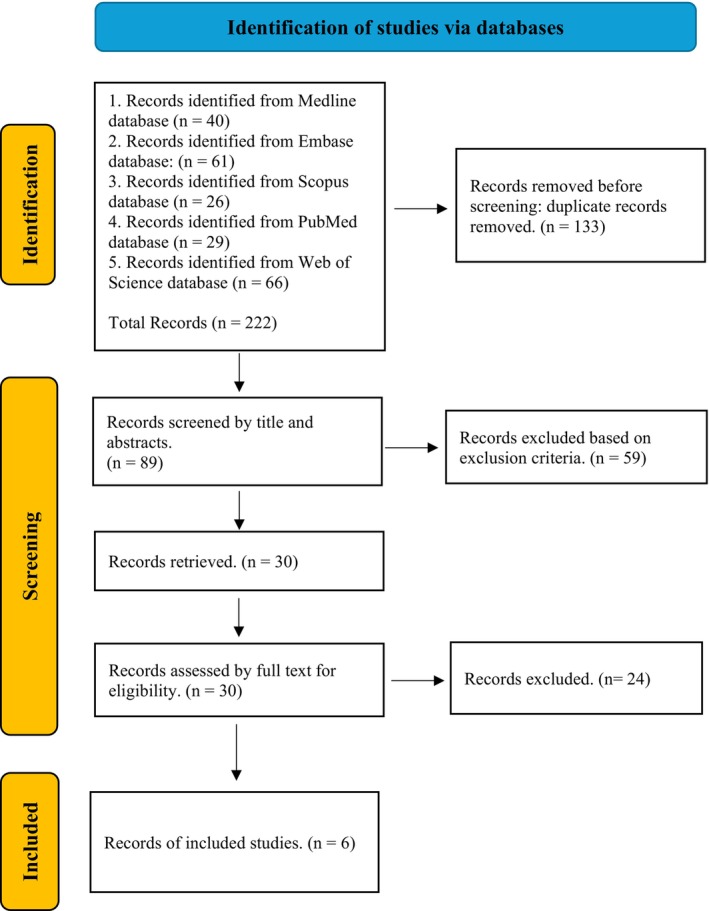
PRISMA flow diagram.

The entire selection process is summarised in Figure 1. The electronic search yielded 222 articles. After removing 89 duplicates, 133 articles remained for the next screening phase. In the first stage, titles and abstracts were reviewed, resulting in the identification of 30 articles. This was followed by a comprehensive full‐text review in the second stage, which excluded 24 studies. The reasons for exclusion were stated in the Supporting Information. As a result, six articles were selected, and their characteristics are presented in Table 2.

**TABLE 2:** Characteristics of included studies.

Title	Author and year	Study type	Sample size	Study setting	Length of study	Study aim
CAD/CAM milled removable complete dentures: time and cost estimation study	Srinivasan et al. 2019	Non‐randomised crossover trial	12	Undergraduate final‐year dental students from the University of Geneva, Switzerland	—	Compared the economic costs of the 2‐visit digital denture protocol with 6‐visit conventional complete denture protocol
A comparison of conventionally versus digitally fabricated denture outcomes in a University Dental Clinic	Clark et al. 2021	Cross‐sectional study	—	Predoctoral students in the University of North Carolina, Adams School of Dentistry	2 years from 2017 to 2019	Compared number of visits and remake rate between conventionally fabricated and digitally fabricated complete dentures
Use of digital tools for preclinical training in complete denture: A pilot study	Porcherot et al. 2024	Randomised crossover trial	210	Third‐year dental students from the Faculty of Dentistry, the University of Paris	—	Assessed the benefit of digital training in conceptual skills acquisition.
Effectiveness of a 3D simulation tool to teach the designing of metal removable partial dentures: A mixed‐method study	Dehurtevent et al. 2023	Randomised controlled trial	43	Third year students at the Faculty of Dental Surgery of the KU Leuven	1 week	Evaluated a 3D‐simulation tool's efficacy for teaching metal removable partial denture (mRPD) design
Comparison of treatment outcomes in digital and conventional complete removable dental prosthesis fabrications in a predoctoral setting	Kattadiyil et al. 2015	Cohort study	15	Third‐ and fourth‐year predoctoral dental students at Loma Linda University School of Dentistry	—	Comparing digitally and conventionally fabricated complete removable dentures in terms of treatment outcomes, patient satisfaction, and dental student preferences
Evaluation on a teaching software for removable partial denture framework design	Luo et al. 2023	Randomised controlled trial	131	Undergraduate students from the Kunming Medical University of China	1 academic year	Evaluated the effect of software teaching on the preclinical teaching of RPD

### 3.1 Description of Studies Included

Of the six articles, two were conducted in the United States, while the others originated from Switzerland, France, Belgium and China. The studies primarily involved third‐year undergraduate dental students and were of short duration. In terms of integrating digital removable dentures into the dental curriculum, four studies focused on digital complete dentures, while two addressed metal partial denture framework design (Table 3). All articles discussed the students' experiences, thereby addressing our second research question, and these findings are summarised in Table 4.

**TABLE 3:** Risk of bias assessed using the Joanna Briggs Institute (JBI) critical appraisal checklist for analytical cross‐sectional studies.

Analytical cross‐sectional study									
Study	Criteria for inclusion defined	Detailed description of subjects and setting	Measurement of exposure valid and reliable	Measurement of a condition done using objective and standard criteria	Confounding factors identified	Statement of strategies to deal with confounding factors	Measurement of outcomes valid and reliable	Appropriate statistical analysis	Overall appraisal
Clark et al. 2021	Yes	Yes	Yes	Yes	No	No	Yes	Yes	Included

**TABLE 4:** Risk of bias assessed using the Joanna Briggs Institute (JBI) critical appraisal checklist for cohort studies.

Cohort studies												
Study	Similar and from the same population groups	Measure of exposures similar to assign people	Measurement of exposure valid and reliable	Confounding factors identified	Statement of strategies to deal with confounding factors	Participants free of the outcome at the start of the study	Measurement of outcomes valid and reliable	Sufficient and long enough follow‐up time	Drop out clearly explained and detailed	Strategies to address incomplete follow‐up	Appropriate statistical analysis	Overall appraisal
Kattadiyil et al. 2015	Unclear	Yes	Yes	No	No	Yes	Yes	No	Not applicable	Not applicable	Yes	Included

### 3.2 Quality Assessment

The study by Clark et al. [25] was evaluated using the checklist for analytical cross‐sectional studies [26] and met most criteria, including clear inclusion criteria, detailed descriptions of subjects and settings, valid and reliable exposure measurements, and appropriate statistical analysis, though it inadequately addressed confounding factors (Table 3). This study was included due to its strong adherence to most appraisal criteria.

Kattadiyil et al. [9] was assessed using the checklist for cohort studies [26]; it reliably measured exposures and ensured participants had no experience of outcome at the start but fell short in managing confounding factors, follow‐up duration, and explaining dropouts (Table 4). Despite these limitations, the study was still included due to its overall adherence to cohort study criteria.

Porcherot et al. [27], Dehurtevent et al. [28], and Luo et al. [29] were evaluated with the checklist for randomised controlled trials [24] and showed strengths in randomisation, baseline similarity, and reliable outcome measurement, but had bias related to administration of intervention or exposure (Table 5). These factors can affect the quality of these studies; nevertheless, they were included due to the limited number of randomised controlled trials presented that met the inclusion criteria of this SR.

**TABLE 5:** Risk of bias assessed using the Joanna Briggs Institute (JBI) critical appraisal checklist for randomised controlled trials.

Randomised controlled trials	Domain	Internal validity bias related to													Overall appraisal
		Selection and allocation			Administration of intervention/exposure			Assessment, detection and measurement of the outcome			Participant retention	Statistical conclusion validity			
	Question no.	1	2	3	4	5	6	7	8	9	10	11	12	13	
	Study														
	Porcherot et al. 2024	Yes	No	Unclear	No	No	No	No	Yes	Yes	Not applicable	No	Yes	Yes	Included
	Dehurtevent et al. 2023	Yes	No	Yes	No	No	No	No	Yes	Yes	Not applicable	No	Yes	Yes	Included
	Luo et al. 2023	Yes	No	Yes	No	No	No	No	Yes	Yes	Not applicable	No	Yes	Yes	Included

Srinivasan et al. [30] were assessed using the checklist for quasi‐experimental studies [31] and demonstrated clear cause‐and‐effect relationships and reliable outcome measurements. Although it had uncertainties in intervention administration, the study was included due to its strong adherence to quasi‐experimental principles (Table 6).

**TABLE 6:** Risk of bias assessed using the Joanna Briggs Institute (JBI) critical appraisal checklist for quasi‐experimental studies.

Non‐randomised crossover trial (quasi‐experimental studies)	Domain	Internal validity bias related to									Overall appraisal
		Temporal precedence	Selection and allocation	Confounding factors	Administration of intervention/exposure	Assessment, detection, and measurement of the outcome			Participant retention	Statistical conclusion validity	
	Question no.	1	2	3	4	5	6	7	8	9	
	Study										
	Srinivasan et al. 2019	Yes	Yes	Yes	Unclear	Yes	Yes	Yes	Not Applicable	Yes	Included

### 3.3 Teaching Digital Removable Complete Denture

The curriculum design of digital removable complete dentures can be described by 2 approaches: didactic training and clinical workflow. Didactic teaching described by Porcherot et al. [27] utilised the ‘Complete Denture’ module of the 3Shape Dental System. The focus was on designing and fabricating custom trays using this 3D‐software, supported by an online tutorial and in‐person demonstrations in class [27].

All clinical workflows mentioned in the selected studies have adopted the AvaDent digital denture system. Kattadiyil et al. [9] and Srinivasan et al. [30] employed a 2‐visit protocol within this system. During the first clinical visit, definitive impressions were made, and jaw relations were recorded using an anatomical measuring device (AMD) provided by AvaDent. In the laboratory, these records were scanned to create virtual models with 3D‐software. An electronic preview of the virtual teeth setup was then sent to students for review before the dentures were milled. The second visit was for inserting the final denture [9, 30]. Clark et al. [25] employed a 4‐visit protocol, integrating conventional fabrication steps into the digital system. The first visit involved taking primary impressions. Students then fabricated custom trays in the lab for definitive impressions taken during the second clinical visit. The third clinical visit was for Wagner try‐in (WTI), allowing students to adjust anterior teeth for aesthetics and function. Once satisfied, the WTI was scanned and proceeded to the fabrication process. The fourth clinical visit was for the final denture insertion [25].

### 3.4 Teaching Digital Removable Metal Partial Framework Design

The educational approach to digital removable partial dentures primarily focuses on the metal framework design process in didactic training. Dehurtevent et al. [28] has implemented a 3D‐simulation tool in RPD lectures, offering scenario‐based learning with 74 clinical cases. This tool allowed students to manipulate designs from various angles, visualise undercut zones on abutment teeth, adjust fulcrum lines and simulate prosthesis movement [28]. On the other hand, Luo et al. [29] created software with similar functions. This software provided students with basic information before attending lectures on RPD design principles. Then, this information was reinforced during lectures and students were required to apply the knowledge in practice by completing assigned tasks following teacher guidance [29].

### 3.5 Educational Experiences

The adoption of digital workflows has received positive feedback from students. With limited clinical experience, undergraduate students benefit from the streamlined procedures, allowing them to fabricate and deliver dentures in just two visits [30]. Students have reported spending less time on each case due to fewer fabrication and review visits, which enables them to practice more cases and improve their skills [25]. Overall, students expressed a strong preference for digital dentures, finding digital workflow easier to perform [9, 25]. Students reported that the ability to save and reuse design files has also facilitated easier remakes of dentures, particularly when addressing unintentional errors [25].

Digital tools used in didactic teaching have shown improvements in the conceptual understanding of the denture design process [27, 28, 29]. For complete dentures, learning through digital tools allows students to better understand laboratory sequences, complementing manual practical sessions [27]. For metal partial denture frameworks, students can visualise the 3D‐spatial structure of specific clinical cases, helping them grasp complex 3D‐structures and reduce design errors [29]. Students reflected those digital tools clarified and enhanced their understanding of theoretical knowledge, such as biomechanics and RPD design principles [28, 29].

From educators' perspectives, using digital tools improves the quality of assessments by enabling them to discern whether students are struggling with motor or conceptual skills [27]. Additionally, a positive outcome is the increased motivation of students due to repeatable, interesting, and clinically relevant exercises [29], along with a higher perceived sense of competence, choice, and interest [28].

Despite these benefits, areas for further improvement of digital tools were indicated. Technical issues with digital tools in didactic teaching are a major concern for students, leading to lower satisfaction and perceived ease of use [27, 28]. Examples include software errors, difficulties in navigation, and limited flexibility of digital tools that restricted students' ability to explore alternative design solutions and test different hypotheses [28]. Another limitation reported is the inability to evaluate design results and receive feedback [29].

Regarding digital workflows, some students reported that digital systems lack the aesthetic trial placements and personalisation provided by traditional methods. They also found it harder to evaluate the function and aesthetics of dentures on digital preview images [9]. However, these issues were only present in the 2‐visit protocol and were addressed in the 4‐visit protocol through the WTI process.

## 4 Discussion

Current literature suggests that the integration of digital denture workflows into dental curricula is still in its early stages, exhibiting significant variability across institutions. This variability is driven by differences in resources, technological access and the specific educational goals of each programme. Our findings indicate that many dental schools favour a hybrid approach that combines digital and conventional workflows, rather than fully transitioning to digital methods. This balanced strategy maximises the benefits of advanced digital technologies while preserving essential practical skills taught through traditional methods. This approach appears crucial in preparing students for the complexities of modern dental practice, where adaptability and proficiency in both techniques are becoming increasingly important.

In most programmes, students are introduced to digital workflows in the third year, after building foundational knowledge through conventional techniques. As outlined in Tables 7 and 8, procedural steps that are often developed digitally include digital impressions, jaw relation using CAD tools, denture design, and fabrication through 3D printing. This allows students to apply clinical reasoning to a digital context while reinforcing core prosthodontic principles. To support learning, many educators advocate for a tandem teaching approach—where digital methods are taught alongside traditional ones. This allows students to critically compare workflows, appreciate the strengths and weaknesses of each method and develop the versatility needed for diverse clinical scenarios. Digital learning also enables students to engage in independent practice at home, such as through treatment planning software and design exercises, increasing exposure to a wider range of simulated patient cases.

**TABLE 7:** Curriculum design of the digital removable dentures.

Title	Author and year	Didactic training	Clinical workflow
CAD/CAM milled removable complete dentures: time and cost estimation study	Srinivasan et al. 2019	N/A	Two visits AvaDent protocol: First visit for taking definitive PVS impressions using a stock tray, recording the jaw relation with an anatomical measuring device, registering the bite, determining the occlusal plane and selecting teeth. Records were scanned into design software to create virtual models, and an electronic preview of the virtual teeth setup was sent to the clinician before milling the dentures. Second visit for denture insertion.
A Comparison of conventionally versus digitally fabricated denture outcomes in a university dental clinic	Clark et al. 2021	N/A	Four visits AvaDent protocol: First visit for primary impression and special tray fabrication. Second visit for definitive impression. Third visit for wagner try‐in. Fourth visit for denture insertion
Use of digital tools for preclinical training in complete denture: A pilot study	Porcherot et al. 2024	Utilised ‘Complete Denture’ module of the 3Shape Dental System for students to design a custom tray using the 3D‐occlusion rim conception tool. An online tutorial and an in‐person demonstration were provided	N/A
Effectiveness of a 3D‐simulation tool to teach the designing of metal removable partial dentures: A mixed‐method study	Dehurtevent et al. 2023	Students used a 3D‐tool which offered scenario‐based learning with 74 clinical cases, enabled them to manipulate designs from different angles, projected undercut zones on abutment teeth, adjusted fulcrum lines and simulated prosthesis movement	N/A
Comparison of treatment outcomes in digital and conventional complete removable dental prosthesis fabrications in a predoctoral setting	Kattadiyil et al. 2015	Students learnt the fabrication conventional denture from their didactic curriculum. They received instructions on the fabrication of digital denture from a 26‐min video prior to the experiment	Two visits AvaDent protocol: First visit for taking definitive impressions, interocclusal records, and teeth selection. Records were scanned and a preview of virtual tooth arrangement was submitted for approval before fabricating the dentures. Second visit for denture insertion
Evaluation on a teaching software for removable partial denture framework design	Luo et al. 2023	Students received training using the software before teacher given the lecture on RPD design principles. Students were asked to complete the assigned design tasks during class, followed by guidance and problem summaries by the teacher	N/A

**TABLE 8:** Educational experiences from students.

Title	Author and year	Positive experiences	Negative experiences
CAD/CAM milled removable complete dentures: time and cost estimation study	Srinivasan et al. 2019	Shorter length of clinical procedure needed for undergraduate students who have limited experience	N/A
A Comparison of Conventionally Versus Digitally Fabricated Denture Outcomes in a University Dental Clinic	Clark et al. 2021	Students spend lesser time on each case due to fewer fabrication visits and post‐operative review Easier for students to remake denture due to saved original design files	N/A
Use of digital tools for preclinical training in complete denture: A pilot study	Porcherot et al. 2024	Improve conceptual skills which enables better learning of laboratory sequences in complement to manual practical sessions Improves the quality of assessment by helping teachers discriminate whether the student is having difficulty acquiring motor and/or conceptual skills	Lower student satisfaction due to software error Potential difficulties in navigating and using the digital tools effectively
Effectiveness of a 3D‐simulation tool to teach the designing of metal removable partial dentures: A mixed‐method study	Dehurtevent et al. 2023	Improve conceptual understanding of mRPD framework design. Students reflected the tool is useful to clarify theoretical lessons and to study specific clinical cases Increase students' motivation due to higher perceived competence, perceived choice and interest. They felt more confident when designing mRPD framework in the clinic	– Lower perceived ease of use as students felt the tool took more time to use than expected – Lower autonomy satisfaction as the tool restricted students to propose their own clinical case or try other design solutions to test their hypothesis, indicating room for improvement
Comparison of treatment outcomes in digital and conventional complete removable dental prosthesis fabrications in a predoctoral setting	Kattadiyil et al. 2015	Students expressed higher preference for digital denture as being easier to perform and no required laboratory work	Students felt that digital protocol lack aesthetic trial placement and less personalised Students reflected difficulty in evaluating digital preview pictures than actual wax trial placement
Evaluation on a teaching software for removable partial denture framework design	Luo et al. 2023	Improve students' learning initiative by providing repeatable, interesting, and clinically relevant exercises Better understanding the RPD 3D‐spatial structure and make theoretical knowledge more visual and intuitive Improve students' 3D‐construction ability which reduces morphological and structural errors when drawing the design scheme	Limitation of the software which could not evaluate the design results and provide timely feedback to students

The preference for hybrid workflow arises from the limitations associated with fully digital methodologies. For instance, physical impressions are often preferred over cross‐arch intraoral scanning due to their mucocompressive properties, which improve tissue adaptation and border moulding to capture functional movement, thereby enhancing retention [32]. Conversely, digital scanning can be prone to inaccuracies caused by various operator and patient factors [33], particularly in critical areas such as the posterior palatal seal and border seal [34]. During the jaw relation step, most digital workflows utilise gothic arch tracing and facebow, typically performed alongside definitive impression‐taking within the same appointment [32]. However, there is a notable lack of evidence demonstrating the clinical superiority of these digital methods. Furthermore, the absence of trial denture placement in some digital workflows may lead to issues such as occlusal relationship errors, insufficient retention, and unsatisfactory aesthetic outcomes [32]. Therefore, the inclusion of a try‐in visit is recommended to facilitate patient‐specific adjustments and enhance the overall quality of the final denture [9, 25, 30, 32, 35].

Currently, available commercial digital denture systems are generally classified into additive (3D‐printing) and subtractive (milling) manufacturing techniques. Subtractive methods, employed by systems such as AvaDent, Ivoclar, Ceramill and Baltic, are more commonly integrated into educational settings due to their clinical reliability and user‐friendliness [36]. Additive techniques, while promising, are less frequently used due to concerns about physical properties, such as dimensional stability and strength [37].

These commercial systems primarily differ in the manufacturer‐produced products they use, and the number of clinical visits required. For instance, the two‐visit workflows of the AvaDent and Baltic systems omit the primary impression to streamline the process and enhance efficiency [10, 38]. The Ivoclar and Ceramill systems involve three‐ to four visits, requiring two impression‐taking procedures to achieve better border capture [38, 39]. Additionally, modified clinical workflows developed, such as the Geneva Protocol and the Functionally Suitable Digital Complete Denture (FSD) protocol, combine the definitive impression‐taking and jaw registration within the same visit. The Geneva Protocol utilises a custom tray with integrated occlusal rims [40], while the FSD protocol employs a closed‐mouth custom tray that resembles the shape of a complete denture which also serves as a trial denture for evaluation [35]. Both protocols are potentially suitable for dental curricula, as they emphasise precision and equip students with essential skills. A significant advantage of these workflows is their manufacturer independence, which may facilitate greater acceptability and a smoother transition for dental programmes.

It is clear that digital technologies enhance clinical efficiency, accuracy, and patient satisfaction [36, 37]. Our findings reveal that the integration of digital workflow has gained considerable preference and positive feedback from both students and educators. Furthermore, it has been shown to improve student competencies, including technical skills, clinical reasoning, and patient‐centred care.

Despite these advances, the clinical application of digital workflows appears to be focused largely on complete dentures. While partial dentures are discussed in the preclinical context, they are notably absent from clinical teaching in the literature reviewed. This may reflect current limitations in the digital design of removable partial dentures–such as digital clasping systems and major connector design–or a lack of validated clinical protocols. If digital workflows remain confined to laboratory stages for both complete and partial dentures, this limits students' clinical exposure to fully digital cases. Further research and curriculum planning are needed to bridge this gap and support the broader clinical implementation of digital technologies.

While digital partial denture workflows are covered in preclinical education, they are less common in clinical teaching, reflecting broader trends in practice. Digital design of frameworks, including clasp assemblies and major connectors, often requires advanced software and laboratory involvement, limiting its use in undergraduate settings. This is similar to complete dentures, where digital tools are used for design, but clinical steps still rely on conventional methods. The gap suggests that more research is required to enhance the digital usage in the construction of partial denture prior to integrating in the educational curriculum.

However, several barriers hinder the widespread adoption of digital workflows in dental education. Financial constraints are a prominent issue, as the initial costs of equipment, software, and ongoing maintenance present a significant burden for many institutions [20]. These costs include not only the acquisition of advanced equipment, but also ongoing expenses related to software updates, equipment maintenance, and the higher costs of materials used in digital fabrication. Additionally, educational institutions often need skilled IT staff to maintain and troubleshoot these digital systems, adding to the financial burden and leading some institutions to prioritise other educational needs over digital denture technology [41].

Infrastructure challenges also complicate the implementation of digital workflows. Upgrading clinical spaces to support digital workflows requires significant investment in renovations and new equipment, and the integration of digital technologies with existing electronic patient records can be logistically complex [41]. These upgrades are essential to fully realise the benefits of digital dentures but can be disruptive and resource‐intensive, particularly for programmes with established infrastructures that are not easily modified. Additionally, integrating digital dentures into existing curricula necessitates careful planning and restructuring, posing difficulties for established programmes with limited flexibility [42].

Regulatory frameworks also play a significant role in the adoption of digital dentures in educational settings. For instance, while the FDA's 510(k) clearance process in the United States has facilitated quicker integration of digital technologies, other regions face stricter regulations that can delay the approval and implementation of new systems [43]. Compliance with existing legal and ethical regulations regarding patient privacy and material safety in digital workflows may lead to delays in regulatory approvals, thereby slowing the adoption of innovative technology in educational settings [44].

Moreover, literature emphasises the importance of validating and standardising digital denture systems before they are widely adopted in dental education. These technologies must undergo rigorous validation to ensure accuracy and reliability before being integrated into dental curricula [17]. Many educators remain cautious about fully transitioning to these new technologies without clear evidence of their long‐term clinical efficacy. This need for validation explains why many institutions hesitate to depart from traditional methods that have been long‐established and consistently reliable [45]. Until these technologies are fully proven, hybrid techniques that blend digital and traditional processes are likely to be the favoured mode of training in many dental schools.

Digital dentures represent a significant advancement in the field of prosthodontics, marking a transformative shift towards more efficient and patient‐centred solutions. Just as conventional dentures faced early challenges that were overcome through innovations in materials and techniques, a similar evolution is anticipated for digital dentures. To effectively integrate these innovations into dental education, it is crucial to adopt a strategic approach that provides comprehensive training in both digital and conventional techniques. By incorporating digital denture methodologies into their curricula and fostering innovation through research initiatives, these institutions can drive meaningful advancements in the field. With the collaborative efforts of manufacturers, dental professionals, and academic institutions, the domain of digital dentures is poised for continued evolution, overcoming existing limitations and ultimately enhancing patient outcomes.

### 4.1 Practical Strategies for Educators

This systematic review suggests that educators should consider hybrid teaching models that combine both conventional and digital workflows, providing students with a more predictable and reliable clinical outcome. Preclinical teaching can incorporate 3D design software and simulation tools to enhance spatial skills and design accuracy. Well‐defined clinical protocols, such as AvaDent or manufacturer‐independent workflows like the Geneva and FSD protocols, offer practical and teachable frameworks. The Geneva protocol may be particularly useful when purchasing external software is expensive. Implementation can begin with simulation settings, offering opportunities for independent practice and feedback. To support successful long‐term adoption, faculty development and staged investment in infrastructure are essential, as integrating digital workflows remains a resource‐intensive process.

## 5 Conclusion

The future of digital dentistry is poised for transformative advancements that could further revolutionise how dental care is delivered and taught. As evidence of the benefits of digital dentures grows, dental schools are likely to increasingly include them in their curricula to ensure students are well‐equipped to leverage these innovations for improved patient care dentistry. This SR demonstrates various institutional approaches to incorporating digital dentures, offering a valuable framework for implementation.

## Author Contributions

All authors contributed to the study and manuscript and have approved the final version.

## Ethics Statement

The authors have nothing to report.

## Conflicts of Interest

The authors declare no conflicts of interest.

## Supporting information


Data S1.

## Data Availability

The authors have nothing to report.

## References

[1] D. A. Felton , “Complete Edentulism and Comorbid Diseases: An Update,” Journal of Prosthodontics 25, no. 1 (2016): 5–20, 10.1111/jopr.12350. 26371954

[2] A. Gupta , D. A. Felton , T. Jemt , and S. Koka , “Rehabilitation of Edentulism and Mortality: A Systematic Review,” Journal of Prosthodontics 28, no. 5 (2019): 526–535, 10.1111/jopr.12792. 29573048

[3] M. T. Kattadiyil and A. AlHelal , “An Update on Computer‐Engineered Complete Dentures: A Systematic Review on Clinical Outcomes,” Journal of Prosthetic Dentistry 117, no. 4 (2017): 478–485, 10.1016/j.prosdent.2016.08.017. 27881317

[4] W. Att , S. Witkowski , and R. Strub, Jr. , Digital Workflow in Reconstructive Dentistry (Quintessence Publishing, 2019).

[5] P. A. Steinmassl , F. Klaunzer , O. Steinmassl , H. Dumfahrt , and I. Grunert , “Evaluation of Currently Available CAD/CAM Denture Systems,” International Journal of Prosthodontics 30, no. 2 (2017): 116–122, 10.11607/ijp.5031. 28267817

[6] S. Shujaat , M. M. Bornstein , J. B. Price , and R. Jacobs , “Integration of Imaging Modalities in Digital Dental Workflows‐Possibilities, Limitations, and Potential Future Developments,” Dento Maxillo Facial Radiology 50, no. 7 (2021): 20210268, 10.1259/dmfr.20210268. 34520239 PMC8474138

[7] M. S. Bilgin , E. N. Baytaroğlu , A. Erdem , and E. Dilber , “A Review of Computer‐Aided Design/Computer‐Aided Manufacture Techniques for Removable Denture Fabrication,” European Journal of Dentistry 10, no. 2 (2016): 286–291, 10.4103/1305-7456.178304. 27095912 PMC4813451

[8] M. Srinivasan , N. Kalberer , N. Fankhauser , M. Naharro , S. Maniewicz , and F. Müller , “CAD‐CAM Complete Removable Dental Prostheses: A Double‐Blind, Randomized, Crossover Clinical Trial Evaluating Milled and 3D‐Printed Dentures,” Journal of Dentistry 115 (2021): 103842, 10.1016/j.jdent.2021.103842. 34637889

[9] M. T. Kattadiyil , R. Jekki , C. J. Goodacre , and N. Z. Baba , “Comparison of Treatment Outcomes in Digital and Conventional Complete Removable Dental Prosthesis Fabrications in a Predoctoral Setting,” Journal of Prosthetic Dentistry 114, no. 6 (2015): 818–825, 10.1016/j.prosdent.2015.08.001. 26412000

[10] P. S. Manoharan , P. R. Wase , and S. Sivakumar , “Challenges and Solutions in Clinical Workflow for the Rehabilitation of Completely Edentulous Patients Through CAD/CAM Dentures: A Case Study,” Cureus 16, no. 3 (2024): e55394, 10.7759/cureus.55394. 38562321 PMC10984337

[11] A. Zandinejad , F. Floriani , W. S. Lin , and A. Naimi‐Akbar , “Clinical Outcomes of Milled, 3D‐Printed, and Conventional Complete Dentures in Edentulous Patients: A Systematic Review and Meta‐Analysis,” Journal of Prosthodontics 33, no. 8 (2024): 736–747, 10.1111/jopr.13859. 38666691

[12] C. Herpel , A. Tasaka , S. Higuchi , et al., “Accuracy of 3D Printing Compared With Milling–A Multi‐Center Analysis of Try‐In Dentures,” Journal of Dentistry 110 (2021): 103681, 10.1016/j.jdent.2021.103681. 33905767

[13] S. F. Iftekar , A. Aabid , A. Amir , and M. Baig , “Advancements and Limitations in 3D Printing Materials and Technologies: A Critical Review,” Polymers 15, no. 11 (2023): 2519. 37299318 10.3390/polym15112519 PMC10255598

[14] X. Sun , L. Meng , Y. Chen , J. Wang , and Q. Wang , “Efficacy and Risk Factors of Traditional Denture Restoration Versus Biofunctional Complete Denture Restoration System,” American Journal of Translational Research 15, no. 7 (2023): 4755–4762. 37560251 PMC10408514

[15] D. R. Prithviraj , H. K. Bhalla , R. Vashisht , K. Sounderraj , and S. Prithvi , “Revolutionizing Restorative Dentistry: An Overview,” Journal Indian Prosthodontic Society 14, no. 4 (2014): 333–343, 10.1007/s13191-014-0351-5. PMC4257941 25489155

[16] L. Lo Russo , K. Zhurakivska , L. Guida , K. Chochlidakis , G. Troiano , and C. Ercoli , “Comparative Cost‐Analysis for Removable Complete Dentures Fabricated With Conventional, Partial, and Complete Digital Workflows,” Journal of Prosthetic Dentistry 131, no. 4 (2024): 689–696, 10.1016/j.prosdent.2022.03.023. 35660258

[17] H. Sri , S. Maiti , and K. Sasanka , “Knowledge, Attitude, and Practice of Digital Dentures Among Dentists,” Journal of Advanced Pharmaceutical Technology & Research 13, no. Suppl 2 (2022): S519–S524, 10.4103/japtr.japtr_186_22. 36798570 PMC9926612

[18] M. M. van der Zande , R. C. Gorter , and D. Wismeijer , “Dental Practitioners and a Digital Future: An Initial Exploration of Barriers and Incentives to Adopting Digital Technologies,” British Dental Journal 215, no. 11 (2013): E21, 10.1038/sj.bdj.2013.1146. 24309814

[19] C. J. Goodacre , B. J. Goodacre , and N. Z. Baba , “Should Digital Complete Dentures be Part of A Contemporary Prosthodontic Education?,” Journal of Prosthodontics 30, no. S2 (2021): 163–169. 10.1111/jopr.13289 33210374

[20] H. Elkassaby , F. Touloumi , W. A. Clark , et al., “A Survey on Utilization and Barriers of Digital Removable Prostheses in the US Dental Education,” Journal of Dental Education 87, no. 12 (2023): 1746–1753, 10.1002/jdd.13372. 37712337

[21] J. J. Kim , J. C.‐C. Yuan , C. Sukotjo , and S. D. Campbell , “Survey of Current Predoctoral Removable Partial Denture Curriculum in the United States,” PRO 3, no. 2 (2021): 119–128, 10.3390/prosthesis3020013.

[22] Y. Ishida , Y. Kuwajima , T. Kobayashi , et al., “Current Implementation of Digital Dentistry for Removable Prosthodontics in US Dental Schools,” International Journal of Dentistry 2022 (2022): 7331185. 35464101 10.1155/2022/7331185 PMC9033361

[23] D. Moher , A. Liberati , J. Tetzlaff , D. G. Altman , and Group P , “Preferred Reporting Items for Systematic Reviews and Meta‐Analyses: The PRISMA Statement,” PLoS Medicine 6, no. 7 (2009): e1000097, 10.1371/journal.pmed.1000097. 19621072 PMC2707599

[24] T. H. Barker , J. C. Stone , K. Sears , et al., “Revising the JBI Quantitative Critical Appraisal Tools to Improve Their Applicability: An Overview of Methods and the Development Process,” JBI Evidence Synthesis 21, no. 3 (2023): 478–493, 10.11124/jbies-22-00125. 36121230

[25] W. A. Clark , B. Brazile , D. Matthews , J. Solares , and I. J. De Kok , “A Comparison of Conventionally Versus Digitally Fabricated Denture Outcomes in a University Dental Clinic,” Journal of Prosthodontics 30, no. 1 (2021): 47–50, 10.1111/jopr.13273. 33058337

[26] E. Aromataris , C. Lockwood , K. Porritt , B. Pilla , and Z. Jordan , “JBI Manual for Evidence Synthesis,” 2024.

[27] A. Porcherot , I. Maniani , M. V. Berteretche , et al., “Use of Digital Tools for Preclinical Training in Complete Denture: A Pilot Study,” European Journal of Dental Education 28, no. 1 (2024): 292–301, 10.1111/eje.12948. 37649263

[28] M. Dehurtevent , J. Duyck , F. Depaepe , S. Vanneste , K. Vandamme , and A. Raes , “Effectiveness of a 3D Simulation Tool to Teach the Designing of Metal Removable Partial Dentures: A Mixed‐Method Study,” European Journal of Dental Education 27 (2023): 1117–1126, 10.1111/eje.12906. 36976773

[29] Z. J. Luo , Y. H. Lin , Y. Yin , T. Zhou , and X. X. Li , “Evaluation on a Teaching Software for Removable Partial Denture Framework Design,” Technology and Health Care 31, no. 5 (2023): 1787–1798, 10.3233/THC-220654. 37125579

[30] M. Srinivasan , M. Schimmel , and M. Naharro , “CAD/CAM Milled Removable Complete Dentures: Time and Cost Estimation Study,” Journal of Dentistry 1, no. 80 (2019): 75–79. 10.1016/j.jdent.2018.09.003 30213557

[31] T. H. Barker , N. Habibi , E. Aromataris , et al., “The Revised JBI Critical Appraisal Tool for the Assessment of Risk of Bias for Quasi‐Experimental Studies,” JBI Evidence Synthesis 22, no. 3 (2024): 378–388, 10.11124/jbies-23-00268. 38287725

[32] K. M. Thu , P. Molinero‐Mourelle , A. W. K. Yeung , S. Abou‐Ayash , and W. Y. H. Lam , “Which Clinical and Laboratory Procedures Should Be Used to Fabricate Digital Complete Dentures? A Systematic Review,” Journal of Prosthetic Dentistry 132, no. 5 (2023): 922–938, 10.1016/j.prosdent.2023.07.027. 37689573

[33] N. Shah , M. Thakur , S. Gill , et al., “Validation of Digital Impressions' Accuracy Obtained Using Intraoral and Extraoral Scanners: A Systematic Review,” Journal of Clinical Medicine 12, no. 18 (2023): 5833, 10.3390/jcm12185833. 37762774 PMC10532392

[34] C. Wang , Y. F. Shi , P. J. Xie , and J. H. Wu , “Accuracy of Digital Complete Dentures: A Systematic Review of In Vitro Studies,” Journal of Prosthetic Dentistry 125, no. 2 (2021): 249–256, 10.1016/j.prosdent.2020.01.004. 32115218

[35] K. Deng , Y. Wang , Y. Zhou , and Y. Sun , “Comparison of Treatment Outcomes and Time Efficiency Between a Digital Complete Denture and Conventional Complete Denture: A Pilot Study,” Journal of the American Dental Association (1939) 154, no. 1 (2023): 32–42, 10.1016/j.adaj.2022.09.016. 36509583

[36] G. O'Donoghue and B. Grufferty , “A Prospective, Single Blind, Crossover, Clinical Trial to Assess the Outcomes of Computer‐Engineered Complete Dentures From Impressions and Intra‐Oral Scans of the Edentulous Arches: A Pilot Study,” 2013.

[37] B. J. Goodacre and C. J. Goodacre , “Additive Manufacturing for Complete Denture Fabrication: A Narrative Review,” Journal of Prosthodontics 31, no. S1 (2022): 47–51, 10.1111/jopr.13426. 35313025

[38] N. Z. Baba , B. J. Goodacre , C. J. Goodacre , F. Muller , and S. Wagner , “CAD/CAM Complete Denture Systems and Physical Properties: A Review of the Literature,” Journal of Prosthodontics 30, no. S2 (2021): 113–124, 10.1111/jopr.13243. 32844510

[39] S. Kanakaraj , H. K. K , and R. Ravichandran , “An Update on CAD/CAM Removable Complete Dentures: A Review on Different Techniques and Available CAD/CAM Denture Systems,” International Journal of Applied Dental Sciences 7, no. 1 (2021): 491–498, 10.22271/oral.2021.v7.i1g.1175.

[40] M. Srinivasan , N. Kalberer , M. Naharro , L. Marchand , H. Lee , and F. Muller , “CAD‐CAM Milled Dentures: The Geneva Protocols for Digital Dentures,” Journal of Prosthetic Dentistry 123, no. 1 (2020): 27–37, 10.1016/j.prosdent.2018.12.008. 31079883

[41] N. U. Zitzmann , L. Matthisson , H. Ohla , and T. Joda , “Digital Undergraduate Education in Dentistry: A Systematic Review,” International Journal of Environmental Research and Public Health 17, no. 9 (2020): 3269, 10.3390/ijerph17093269. 32392877 PMC7246576

[42] P. Maragliano‐Muniz and E. D. Kukucka , “Incorporating Digital Dentures Into Clinical Practice: Flexible Workflows and Improved Clinical Outcomes,” Journal of Prosthodontics 30, no. S2 (2021): 125–132, 10.1111/jopr.13277. 33128422

[43] P. Shah , O. Olavarria , N. Dhanani , et al., “The Food and Drug Administration's (FDA'S) 510(k) Process: A Systematic Review of 1000 Cases,” American Journal of Medicine 136, no. 2 (2023): 172–178, 10.1016/j.amjmed.2022.09.006. 36170936

[44] I. J. Borges do Nascimento , H. Abdulazeem , L. T. Vasanthan , et al., “Barriers and Facilitators to Utilizing Digital Health Technologies by Healthcare Professionals,” NPJ Digital Medicine 6, no. 1 (2023): 161, 10.1038/s41746-023-00899-4. 37723240 PMC10507089

[45] S. A. Brownstein , A. Murad , and R. J. Hunt , “Implementation of New Technologies in U.S. Dental School Curricula,” Journal of Dental Education 79, no. 3 (2015): 259–264. 25729019

