# Supplementary material for: Digital Removable Denture Workflows in Dental Education: A Systematic Review and Curriculum Development Exploration
Source: Eur J Dent Educ. 2025 Jun 17;30(2):547–60. doi: 10.1111/eje.70001 (PMC13090417; doi:10.1111/eje.70001)
Supplement: Supplementary file 1 — Data S1. [file EJE-30-547-s001.docx]

# **APPENDIX 1. Electronic search strategies**

**MEDLINE**

1. dentures/ or denture, complete/ or denture, partial/

2. (denture* or “complete denture*” or “partial denture*”).mp.

3. computer-aided design/ or printing, three-dimensional/

4. (digital or CAD or CAM or CAD-CAM or “computer aided design*” or “computer aided manufactur*” or “computer assisted design*” or “computer assisted manufactur*” or “3D print*” or milling).mp.

5. Education, Dental/

6. (“dent* curricul*” or “dent* teach*” or “dent* educa*” or “dent* school*” or “dent* student*”).mp.

7. 1 or 2

8. 3 or 4

9. 5 or 6

10. 7 and 8 and 9

11. limit 10 to (English language and yr=”2010 - Current”)

**EMBASE**

1. removable partial denture/ or partial upper denture/ or partial lower denture/ or denture/ or complete lower denture/ or complete denture/ or partial denture/ or complete upper denture/

2. (denture* or “complete denture*” or “partial denture*”).mp.

3. computer aided design/ or “computer aided design/computer aided manufacturing”/ or rapid prototyping/ or three dimensional computer aided design/ computer aided manufacturing/

4. (digital or CAD or CAM or CAD-CAM or “computer aided design*” or “computer aided manufactur*” or “computer assisted design*” or “computer assisted manufactur*” or “3D print*” or milling).mp.

5. dental education/

6. (“dent* curricul*” or “dent* teach*” or “dent* educa*” or “dent* school*” or “dent* student*”).mp.

7. 1 or 2

8. 3 or 4

9. 5 or 6

10. 7 and 8 and 9

11. limit 10 to (English language and yr=”2010 - Current”)

**PUBMED**

(( denture* [Title/Abstract] OR “complete denture*” [Title/Abstract] OR “partial denture*” [Title/Abstract]) AND (digital [Title/Abstract] OR CAD-CAM [Title/Abstract] OR CAD/CAM [Title/Abstract] OR “computer aided design” [Title/Abstract] OR “computer assisted design” [Title/Abstract] OR “computer assisted manufacturing” [Title/Abstract] OR “computer aided manufacturing” [Title/Abstract])) AND (“dental curricul*” [Title/Abstract] OR “dental teach*” [Title/Abstract] OR “dental educat*” [Title/Abstract] OR “dental school” [Title/Abstract] OR “dental student*” [Title/Abstract]) Filters: English

**SCOPUS**

( TITLE-ABS-KEY ( denture* OR "complete denture*" OR "partial denture*" ) AND TITLE-ABS-KEY ( "dent* curricul*" OR "dent* teach*" OR "dent* educat*" OR "dent* school*" OR "dent* student*" ) AND TITLE-ABS-KEY ( digital OR cad/cam OR cad-cam OR "computer aided design*" OR "computer aided manufactur*" OR "computer assisted design*" OR "computer assisted manufactur*" OR "3D print*" OR milling ) ) AND PUBYEAR > 2010 AND PUBYEAR < 2025 AND ( LIMIT-TO ( LANGUAGE , "English" ) )

**WEB OF SCIENCE**

( TOPIC (denture* OR “complete denture*” OR "partial denture*") AND TOPIC ("dent* curricul*" OR "dent* teach*" OR "dent* educat*" OR "dent* school*" OR "dent* student*") AND TOPIC (digital OR cad/cam OR cad-cam OR "computer aided design*" OR "computer aided manufactur*" OR "computer assisted design*" OR "computer assisted manufactur*" OR "3D print*" OR milling) AND INDEX DATE (2010-01-01 to 2024-04-04)

# **APPENDIX 2. Table of excluded studies**

| **Title** | **Author** | **Reason for Exclusion** |
| --- | --- | --- |
| Development of a thematic learning object for removable partial denture teaching | (Alves et al., 2014) | It focuses on digitalisation of education delivery rather than digitalisation of RPD workflow. |
| Implementation of New Technologies in US Dental School Curricula. Journal of Dental Education | (Brownstein et al., 2015) | Discusses various technologies including fixed pros, endo, and radiology; only a portion addresses digital denture. |
| Study of digital denture systems among dental students. Drug Invention Today | (Bhaskar et al., 2020) | Conducts a survey on students' awareness toward CAD/CAM, without specific focus on digital denture. |
| Survey of Current Predoctoral Removable Partial Denture Curriculum in the United States | (Kim et al., 2021) | Not involving details of curriculum design |
| Current trends in complete denture education in undergraduate dental colleges of Pakistan. Journal of the Pakistan Medical Association | (Kumar et al., 2023) | Focuses on conventional methods for removable denture, without addressing digital approaches. |
| Use of Digital Technology to Improve Objective and Reliable Assessment in Dental Student Simulation Laboratories. Journal of Dental Education | (Miyazono et al., 2019) | Discusses fixed pros materials, without mention of digital denture. |
| Knowledge, Awareness and Practices of the use of Digital Technology in Dentistry among Postgraduate Students and Dental Practitioners in India: A Cross-sectional Study [Article]. Journal of Clinical and Diagnostic Research | (Nayakar et al., 2022) | Focuses on CAD/CAM but lacks specific relevance to removable pros. |
| Using CAD/CAM technology to create a 10-unit zirconia fixed partial denture--a UTHSCSA dental school case report [Case Reports Journal Article]. | (Zimmermann et al., 2013) | Limited to fixed pros materials. |
| A questionnaire on the use of digital denture impressions in a preclinical setting [Article]. International journal of computerized dentistry | (Wegner et al., 2017) | Focuses on scanning for crowns prep for fixed pros only. |
| Implementing a new curriculum for computer-assisted restorations in prosthetic dentistry [Article]. European Journal of Dental Education | (Schweyen et al., 2018) | Concentrates on fixed prosthodontics, lacking relevance to removable dentures. |
| A model of blended learning in a preclinical course in prosthetic dentistry. Journal of Dental Education. | (Reissmann et al., 2015) | Limited to e-learning for lectures, without addressing clinical aspects, design, or workflow. |
| Digital Undergraduate Education in Dentistry: A Systematic Review. Int J Environ Res Public Health | (Zitzmann et al., 2020) | Does not mention curriculum design/workflows for removable dentures. |
| Investigating the Application of New Technologies in Dental Education: A Systematic Review [Review]. Acta Medica Iranica | (Yazdanpanahi et al., 2022) | Discusses overall technology without specific focus on denture technology. |
| Should Digital Complete Dentures Be Part of a Contemporary Prosthodontic Education? | (Goodacre et al., 2021) | Review article |
| Current challenges for 3D printing complete dentures: experiences from a multi-centre clinical trial | (Osnes et al., 2023) | Not involving students |
| Economic and Clinical Impact of Digitally Produced Dentures | (Smith et al., 2021) | Not involving students |
| Teaching removable partial denture design: 'METACIEL', a novel digital procedure | (Bonnet et al., 2018) | A letter to the editor |
| Enhancing Student Learning of Removable Prosthodontics Using the Latest Advancements in Virtual 3D Modelling | (Mahrous & Schneider, 2019) | A demonstration of 3D software without specific focus on curriculum design |
| A survey on utilization and barriers of digital removable prostheses in the US dental education. Journal of Dental Education, | (Elkassaby et al., 2023) | Not involving details of curriculum design |
| Current Implementation of Digital Dentistry for Removable Prosthodontics in US Dental Schools | (Ishida et al., 2022) | Not involving details of curriculum design |
| A new digital system to generate and draw framework design of removable partial dentures | (Abdulhadi et al., 2013) | A technical report |
| Teaching the Design and Fabrication of RPD Frameworks with a Digital Workflow: A Preclinical Dental Exercise | (Mahrous & El-Kerdani, 2020) | Small sample size |
| Denture teeth arrangement using a web-based digital software program: Taking preclinical dental education to another level | (Turkyilmaz & Wilkins, 2021) | A review article |
| Digital Denture Fabrication in Pre- and Postdoctoral Education: A Survey of U.S. Dental Schools | (Fernandez et al., 2016) | Not involving details of curriculum design |
